# 2502. Best Practice Alert for Hepatitis C Virus Screening Demonstrates Importance of Reminders during Disruptive Times (COVID-19)

**DOI:** 10.1093/ofid/ofad500.2120

**Published:** 2023-11-27

**Authors:** Alec R Mason, A L exander P Radunsky, Stephanie M Reyes, Jennifer McBryde, Mamta K Jain

**Affiliations:** UT Southwestern Medical Center, Dallas, Texas; Ut Southwestern, Dallas, Texas; University of Texas Southwestern Medical Center, Dallas, Texas; UT Southwestern Medical Center, Dallas, Texas; UT Southwestern Medical Center, Dallas, Texas

## Abstract

**Background:**

In 2015, a Best Practice Alert (BPA) was implemented into our electronic health record that prompts ordering of Hepatitis C Virus (HCV) antibody (Ab) screens for unscreened patients born between 1945-1965 (baby boomers: BB). The BPA begins the first step of the three-step care cascade, after which follow-up is needed for RNA testing if Ab-positive and HCV clinic visit if RNA-positive (figure 1). Prior studies found a 3.5-fold increase in HCV Ab screening after BPA implementation. We sought to understand the BPA’s role in cascade navigation during the COVID-19 pandemic.

**Figure:**
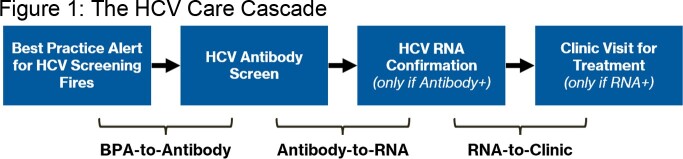

Initial BPA is triggered, which leads to subsequent steps including HCV antibody testing, HCV RNA testing, and ending with a clinic visit for treatment initiation.

**Methods:**

This retrospective study included BB receiving their first BPA between 7/1/18-12/31/19 (pre-pandemic: PP) and 1/1/20-7/1/21, (intra-pandemic: IP), excluding patients with a death date ≤ 180 days after their BPA. Patients were followed for 180 days for each step and were marked unsuccessful if it was not completed. Successful navigation (SN) was defined as completing all steps or testing negative at any step. Hazard ratios for completion of each step were calculated via Cox Proportional Model. Clinical and demographic predictors of SN were examined via multivariable logistic regression in each cohort.

**Results:**

Overall, 14,236 unscreened BB had a BPA (8,090 PP, 6,146 IP) fire during the study periods. The cohorts were similar, except for an increase in share with county financial assistance and those cared for by advanced practice providers in the IP cohort (table 1). An increase in HCV Ab screening (aHR 1.31 [95% CI: 1.25, 1.38]) and decreases in HCV RNA testing (aHR 0.79 [0.65, 0.96]) and clinic visits (aHR 0.43 [0.28, 0.66]) were seen in the IP cohort (figures 2A, B, and C). Hispanic patients had increased odds (aOR: 1.25 [1.02, 1.54]) of SN in the IP cohort (figures 3A and B). Compared to family medicine, those cared for in geriatric clinics had lower odds (aOR 0.75 [0.62, 0.91]) of SN in the IP cohort. Lower odds were also seen for those with Charlson Index ≥ 2 (aOR 0.85 [0.74, 0.98]) compared to 0.

**Table 1.**
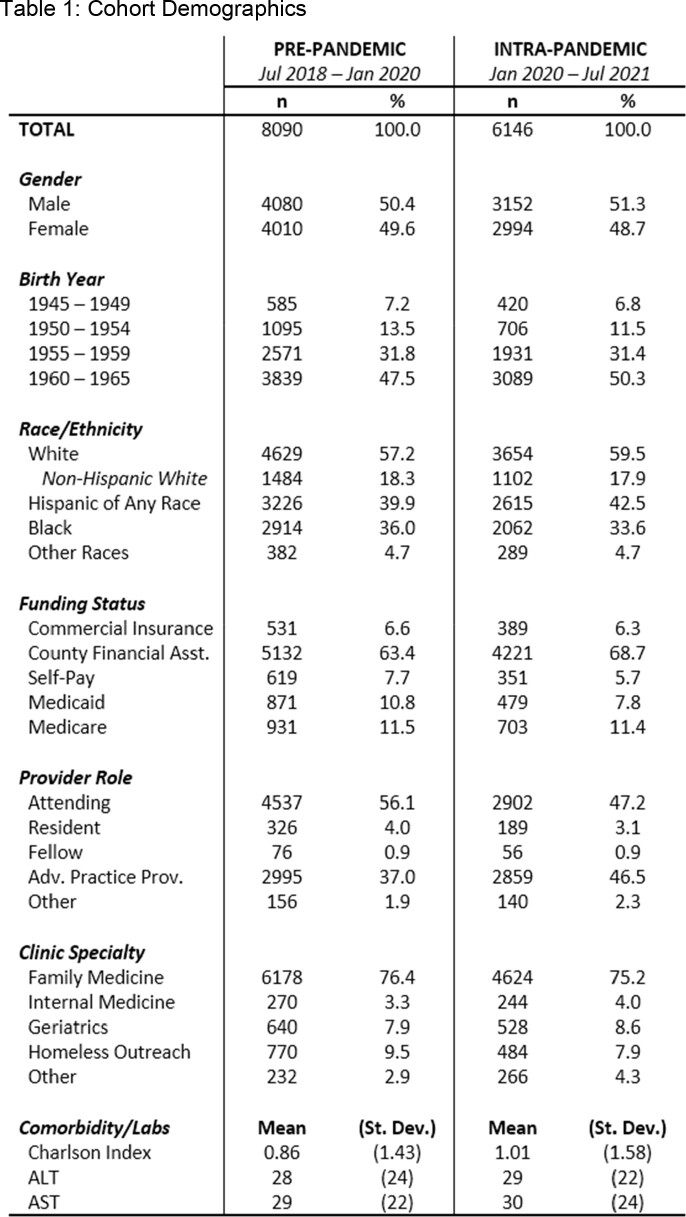
Cohort Demographics.

Baseline demographic, comorbidity, and provider factors for the pre-pandemic and intra-pandemic cohorts.

**Figure 2.**
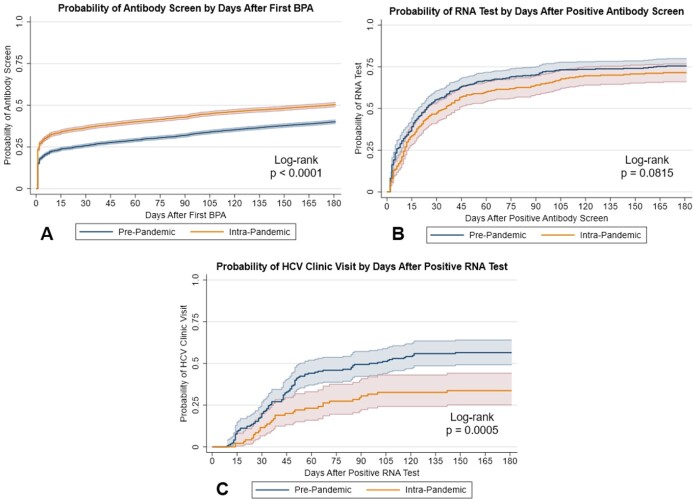
Survival-Time Analysis of the HCV Care Cascade. Kaplan-Meier curves with 95% confidence intervals depicting time to completion by day after step initiation in each cohort. Data for the HCV antibody screening (A), HCV RNA testing (B), and liver clinic visit (C) steps are each shown separately. Log-rank test p-values are shown.

**Figure 3.**
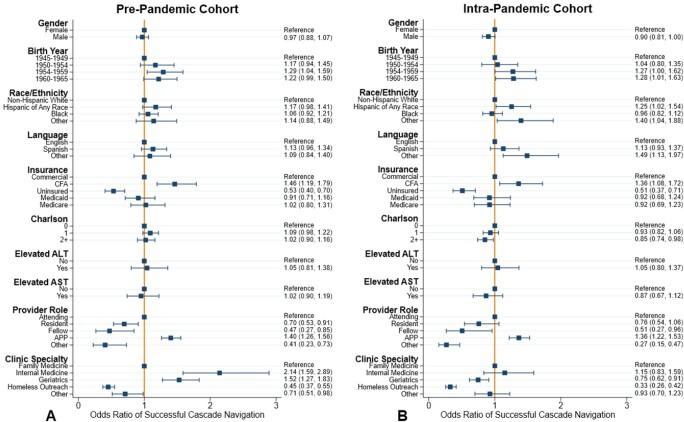
Logistic Regression Forest Plots

Forest plots showing the adjusted odds ratios and 95% confidence intervals for successful cascade navigation in the pre-pandemic (A) and intra-pandemic (B) cohorts. Odds ratio less than 1 indicates lower odds of successful navigation during that period. Odds ratio greater than 1 indicates higher odds of successful navigation.

**Conclusion:**

The BPA increased the durability of HCV Ab screening rates during the pandemic compared to other steps in the cascade. Disparities in completing all steps of the cascade persisted and likely worsened in some groups during the pandemic. Overall, electronic reminders lessen impacts of disruptions in healthcare services.

**Disclosures:**

**Mamta K. Jain, MD, MPH**, Gilead Sciences: Grant/Research Support|Laurent: Grant/Research Support

